# Combined Biomarker Approach Using Extracellular Vesicles, Donor-Derived Cell-Free DNA, and Donor-Specific Antibodies for Monitoring Renal Allograft Function: A Narrative Review

**DOI:** 10.3390/medicina62040664

**Published:** 2026-03-31

**Authors:** Kumar Digvijay, Henrik Birn

**Affiliations:** Department of Renal Medicine, Aarhus University Hospital, 8200 Aarhus N, Denmark

**Keywords:** renal transplantation, extracellular vesicles, donor-derived cell-free DNA, donor-specific antibodies, allograft rejection, liquid biopsy, biomarkers, transplant monitoring

## Abstract

*Background and Objectives:* Renal transplantation is the optimal treatment for end-stage renal disease, yet long-term allograft survival remains threatened by immune-mediated injury and chronic nephropathy. Conventional monitoring using serum creatinine and protocol biopsy suffers from limited sensitivity for early, subclinical injury. Liquid biopsy-based biomarkers offer a non-invasive alternative. *Materials and Methods:* We conducted a systematic narrative review of studies published between January 2010 and December 2024, identified through PubMed, Scopus, and Web of Science. *Results:* Extracellular vesicles carry injury-specific molecular cargo reflecting the biological state of tubular, glomerular, and endothelial cells; urinary EV CXCL9 protein and exosomal CD3ε mRNA have demonstrated AUC values of 0.81–0.88 for the detection of T-cell-mediated rejection. Donor-derived cell-free DNA quantifies global graft cell death; the FDA-cleared AlloSure assay achieves an AUC of 0.74 and NPV of 84% at the validated ≥1.0% threshold established in the DART trial. Donor-specific antibodies—particularly complement-fixing C1q-positive DSAs—confer markedly inferior 5-year graft survival compared with DSA-negative recipients (54% versus 93%). Multi-biomarker panels integrating all three modalities yield AUCs of 0.88–0.94 and NPVs of 91–95%. *Conclusions:* The integration of EV, ddcfDNA, and DSA monitoring into a unified surveillance framework offers a clinically meaningful advance over creatinine-based monitoring. Prospective randomized trials confirming improvement in long-term allograft survival will be the critical next step.

## 1. Introduction

Kidney transplantation is the ultimate treatment for patients with end-stage renal disease (ESRD), providing better survival and quality of life compared with maintenance dialysis [1]. Despite substantial advances in immunosuppressive pharmacology, approximately 20–30% of renal allografts are lost within 10 years, predominantly due to antibody-mediated rejection (ABMR), T-cell-mediated rejection (TCMR), or progressive interstitial fibrosis and tubular atrophy (IFTA) [2,3]. The clinical and economic burden of graft loss is considerable: return to dialysis confers an estimated 50% mortality within 2 years in patients over 60 years of age, and re-transplantation carries substantially higher immunological risk [4].

Routine post-transplant follow-up still highly depends on the serum creatinine and estimated glomerular filtration rate (eGFR), followed by protocol biopsies at predefined post-transplant intervals. However, serum creatinine is a late and intrinsically insensitive indicator of parenchymal damage—measurable elevations emerge only after a substantial proportion of functional nephron mass has already been irreversibly lost, meaning subclinical injury may have been ongoing for weeks or months before any detectable signal appears [5]. Protocol biopsy, while providing direct histological information according to the Banff classification criteria, carries procedural risks including hemorrhage and graft loss in approximately 0.1% of procedures, and is subject to sampling error given the focal, heterogeneous distribution of rejection lesions [6].

The concept of ‘liquid biopsy’—the detection and quantification of circulating analytes that reflect tissue injury—has transformed oncological diagnostics and is increasingly applied in solid organ transplantation [7]. Three biomarker classes have emerged as the most clinically relevant and technically mature: (1) extracellular vesicles (EVs), nanoscale membrane-bound particles released by all cell types whose cargo reflects the biological state of their cell of origin; (2) donor-derived cell-free DNA (ddcfDNA), short nuclear DNA fragments released by dying graft cells into recipient plasma; and (3) donor-specific antibodies (DSAs), immunoglobulins directed against donor human leukocyte antigen (HLA) molecules that mediate humoral alloimmune injury. Each modality captures a distinct dimension of allograft pathophysiology, and converging evidence suggests that their integration into multi-marker panels provides diagnostic accuracy substantially superior to that of any single biomarker [8,9].

This narrative review aims to: (a) describe the biological basis and analytical methods for each biomarker class; (b) critically appraise clinical evidence from landmark trials and observational cohorts; (c) synthesize evidence for combined multi-biomarker monitoring strategies; and (d) provide a practical framework for clinical implementation and identify priorities for future research.

## 2. Materials and Methods

### 2.1. Literature Search Strategy

We performed a comprehensive electronic literature search of PubMed/MEDLINE, Scopus, and Web of Science from January 2010 to December 2024. The following search terms were used in combination using Boolean operators (AND/OR): ‘extracellular vesicles’, ‘exosomes’, ‘microvesicles’, ‘donor-derived cell-free DNA’, ‘ddcfDNA’, ‘donor-specific antibodies’, ‘HLA antibodies’, ‘renal transplantation’, ‘kidney allograft’, ‘allograft rejection’, ‘liquid biopsy’, ‘transplant monitoring’, and ‘biomarkers’. Reference lists of identified articles and relevant review papers were manually screened for additional studies.

### 2.2. Inclusion and Exclusion Criteria

Studies were included if they: (1) involved adult or pediatric human kidney transplant recipients; (2) evaluated EVs, ddcfDNA, DSAs, or combinations thereof as diagnostic or monitoring biomarkers; (3) reported quantitative performance metrics (sensitivity, specificity, AUC, NPV, or PPV); and (4) were published in peer-reviewed journals in English. Studies were excluded if they were: conference abstracts without full-text publication, animal studies, or single case reports. For combined biomarker studies, only those evaluating at least two of the three modalities were included in the synthesis. A total of 47 studies met the final inclusion criteria and are cited throughout this review.

## 3. Results

### 3.1. Extracellular Vesicles (EVs)

#### 3.1.1. Biology and Classification

Extracellular vesicles comprise several subtypes that differ in origin and size. Exosomes arise from the endosomal system and are typically 40–150 nm, and microvesicles form by outward budding of the plasma membrane and range from roughly 100 to 1000 nm, while apoptotic bodies are larger fragments released during programmed cell death [10]. Extracellular vesicles carry a diverse molecular payload—including proteins, lipids, and nucleic acids—whose composition directly reflects the physiological or pathological state of the parent cell. EV secretion is regulated by intracellular calcium flux, sphingomyelinase-driven ceramide pathways, and actin cytoskeletal dynamics, with release markedly amplified by hypoxia, oxidative stress, and inflammatory cytokine signaling [11,12].

The standardization of EV studies is guided by the MISEV2023 framework, which recommends demonstrating vesicle size and concentration using methods such as nanoparticle tracking analysis, confirming morphology by electron microscopy, and verifying vesicle identity through the detection of canonical tetraspanins including CD9, CD63, and CD81 [13].

#### 3.1.2. EV Cargo as Rejection Biomarkers

Urinary EVs offer a uniquely direct window into intrarenal biology because they are shed locally by tubular epithelial, glomerular, and endothelial cells, thereby sampling the allograft microenvironment in real time. Suthanthiran et al., in the landmark multicenter CTOT-04 trial enrolling 485 kidney allograft recipients, demonstrated that a urinary cell three-gene mRNA signature comprising CD3ε, CXCL10 (IP-10), and 18S rRNA was diagnostic of T-cell-mediated rejection (TCMR), with the diagnostic score rising detectably in the weeks preceding creatinine elevation; the overall AUC for TCMR detection was approximately 0.85 [14].

Complementary evidence comes from EV proteomics. Sigdel et al. applied mass spectrometry-based quantitative proteomics to the urinary exosomal fraction from biopsy-matched kidney transplant recipients and identified 11 proteins significantly enriched in patients with acute rejection, including inflammatory mediators functionally linked to cytotoxic T-lymphocyte activity [15]. Separately, urinary CXCL9 protein—an interferon-gamma-induced chemokine produced by tubular cells under alloimmune stimulation—has been validated as a robust rejection biomarker across multicenter cohorts, with AUC values of approximately 0.81–0.85 for biopsy-confirmed acute rejection [16].

For antibody-mediated rejection, endothelial-derived EVs bearing complement fragments (C4d, C3d) have been identified as non-invasive surrogates for biopsy-proven C4d deposition [17]. Platelet-derived microvesicles expressing HLA-DR are elevated in ABMR and correlate with complement-fixing DSA [18]. MicroRNA cargo analysis has revealed that miR-21-5p and miR-142-3p are differentially expressed in urinary EVs during rejection versus stable function, with miR-142-3p specifically upregulated during immune activation [19]. Urinary EVs also reflect non-immunological graft injury: aquaporin-1- and aquaporin-2-expressing vesicles are reduced in delayed graft function and chronic tubular atrophy, reflecting loss of differentiated tubular function [20].

#### 3.1.3. Analytical Methodologies

EV isolation from urine employs differential ultracentrifugation (dUC, reference method), size exclusion chromatography (SEC), precipitation-based commercial kits, or microfluidic platforms. While dUC delivers acceptable vesicle purity, its throughput is limited and yield relatively low. SEC preserves native vesicle architecture more effectively and substantially reduces contamination from co-pelleted protein aggregates, though at greater cost and processing time. Downstream cargo analysis platforms include data-independent acquisition mass spectrometry proteomics, small RNA sequencing, multi-parameter flow cytometry, and digital droplet PCR (ddPCR) for nucleic acid quantification. Pre-analytical variables—urinary osmolality, centrifugation protocols, storage temperature, and freeze–thaw cycles—significantly affect EV yield and cargo integrity, representing the primary barrier to clinical standardization [21].

#### 3.1.4. Limitations and Confounders

Despite their promise, EV-based biomarkers are subject to important biological and analytical limitations. Biologically, EVs are released by virtually all cell types, meaning that urinary or plasma EV preparations inevitably contain a mixture of donor-derived, recipient immune cell-derived, and systemically circulating vesicles; attributing a specific EV signal unambiguously to the allograft remains technically challenging in the absence of validated donor-specific labeling strategies. Inflammatory conditions unrelated to rejection—including urinary tract infections, systemic autoimmune flares, or calcineurin inhibitor-induced tubular stress—can independently upregulate EV release and alter cargo composition, potentially generating false-positive rejection signals [12,21]. Pre-analytical confounders are substantial: the urinary osmolality at the time of collection, centrifugation speed and duration, storage temperature, and number of freeze–thaw cycles all critically affect both the EV yield and cargo integrity, contributing to inter-laboratory variability that currently precludes direct cross-study comparison. The absence of a standardized, clinical-grade EV isolation protocol—despite the MISEV2023 framework providing research guidance [13]—means that no EV assay has yet received regulatory clearance, and multi-site reproducibility remains unestablished. Finally, distinguishing donor-derived from recipient-derived EVs within a mixed urinary sample requires sophisticated labeling or sequencing approaches not yet feasible in routine clinical workflows.

### 3.2. Donor-Derived Cell-Free DNA (ddcfDNA)

#### 3.2.1. Biological Basis and Analytical Platforms

Cell-free DNA (cfDNA) comprises short, mononucleosome-length (~165 bp) DNA fragments released into the circulation primarily through apoptosis and necrosis. In kidney transplant recipients, a fraction of plasma cfDNA originates from donor graft tissue and can be distinguished from recipient-derived cfDNA based on genetic polymorphisms. Under stable conditions, ddcfDNA constitutes approximately 0.2–1.0% of total plasma cfDNA; during graft injury and cell death, this fraction rises substantially [22]. The landmark proof-of-concept study by Snyder et al. (2011) first demonstrated that donor-organ-derived DNA was detectable in recipient plasma and correlated with rejection episodes, establishing the biological foundation for this biomarker class [23].

Two FDA-cleared platforms are commercially available for clinical ddcfDNA testing. AlloSure (CareDx Inc., Brisbane, CA, USA) employs a targeted SNP-based next-generation sequencing approach interrogating 266 highly polymorphic SNPs and requires no prior donor or recipient genotyping, reporting ddcfDNA as a fractional percentage of total cfDNA [24]. Prospera (Natera Inc., Austin, TX, USA) utilizes a massively multiplexed PCR approach targeting 13,962 SNPs, similarly requiring no separate donor or recipient genotyping, and reports both fractional and absolute ddcfDNA quantification [25]. Both assays require blood collection into cfDNA-stabilizing tubes (Streck Cell-Free DNA BCT, La Vista, NE, USA) and plasma processing within specified time windows to prevent leukocyte lysis and consequent contamination of plasma cfDNA [24,25].

#### 3.2.2. Clinical Evidence

The pivotal DART (Diagnosing Active Rejection in Kidney Transplant Recipients) prospective multicenter study (*n* = 102 recipients, 107 biopsies) established the clinical performance of ddcfDNA. A threshold of ≥1.0% ddcfDNA identified active rejection (ABMR and TCMR) with a sensitivity of 59%, specificity of 85%, AUC of 0.74, and NPV of 84% [26]. Importantly, ddcfDNA performed significantly better for ABMR and mixed rejection than for TCMR alone, reflecting the greater degree of complement-mediated and NK cell-mediated endothelial injury in antibody-mediated processes. A subsequent analysis by Oellerich et al. using absolute ddcfDNA quantification (copies/mL) demonstrated superior discrimination of ABMR from non-rejection injury states compared with fractional measurement [27].

The prospective multicenter Kidney Allograft Outcomes AlloSure Registry (KOAR), enrolling 1743 recipients across 56 centers, demonstrated that serial ddcfDNA surveillance significantly improved biopsy rejection yield, with elevated ddcfDNA associated with a six-fold increase in rejection detection on surveillance biopsies and a four-fold increase on for-cause biopsies; furthermore, ddcfDNA elevations were detectable up to four months before biopsy-proven ABMR [28]. The ADMIRAL multicenter observational study (*n* = 1092 across 7 centers) demonstrated that elevation of ddcfDNA ≥ 0.5% was significantly correlated with clinical and subclinical allograft rejection, and predicted de novo DSA formation a median of 91 days in advance, and that persistently elevated ddcfDNA predicted a >25% decline in eGFR [29]. In pediatric recipients, ddcfDNA monitoring has been validated as a useful surveillance strategy with performance characteristics broadly consistent with those observed in adult cohorts [30].

#### 3.2.3. Limitations and Confounders

A critical limitation of ddcfDNA is its inability to distinguish immunological from non-immunological causes of graft cell death. BK polyomavirus nephropathy (BKPyVN), ischemia–reperfusion injury, calcineurin inhibitor nephrotoxicity, and urinary tract infections all elevate ddcfDNA independently of rejection, limiting the positive predictive value (PPV) to approximately 40–50% in unselected populations [29]. Fractional quantification is additionally confounded by the total cfDNA background from recipient conditions (infections, malignancy, trauma). The emerging approach of cfDNA methylation profiling—exploiting tissue-specific methylation patterns to identify the tissue of origin of elevated cfDNA—holds promise for improving diagnostic specificity in this context [31].

### 3.3. Donor-Specific Antibodies (DSAs)

#### 3.3.1. Immunobiology of DSA-Mediated Injury

Donor-specific antibodies are immunoglobulins—predominantly IgG—directed against donor HLA class I (HLA-A, -B, -C) and class II (HLA-DR, -DQ, -DP) antigens. DSAs may be pre-formed (present at transplantation due to prior sensitization via transfusion, pregnancy, or previous transplant) or de novo (arising post-transplant through T-cell-dependent B-cell activation) [32]. De novo DSA (dnDSA) formation is strongly associated with subclinical and clinical ABMR; longitudinal registry data demonstrate that dnDSA is detectable at a mean of 4–5 years post-transplant in nonsensitized recipients, with subclinical antibody-mediated injury present in the majority of dnDSA-positive patients even in the absence of graft dysfunction [33].

Mechanistically, DSA mediates graft injury through complement-dependent and complement-independent pathways. Complement-activating DSAs (C1q-binding, C3d-binding) trigger the classical complement cascade, generating C3a/C5a anaphylatoxins and the C5b-9 membrane attack complex (MAC), causing direct endothelial cell lysis and peritubular capillaritis. Complement-independent injury occurs via Fc receptor signaling: natural killer (NK) cell-mediated antibody-dependent cellular cytotoxicity (ADCC), macrophage FcγR activation, and neutrophil degranulation all contribute to chronic endothelial damage and progressive vascular intimal proliferation [32,34]. The clinical phenotype of DSA-mediated chronic ABMR includes progressive proteinuria, transplant glomerulopathy on biopsy, the multilayering of peritubular capillary basement membranes, and progressive eGFR decline.

#### 3.3.2. DSA Characterization and Risk Stratification

Modern DSA detection and characterization utilizes solid-phase single antigen bead (SAB) Luminex technology, which enables the detection of antibodies at mean fluorescence intensities (MFIs) as low as 500–1000 MFI and identification of the specific HLA target. Risk stratification incorporates multiple DSA characteristics: MFI strength (high-risk typically >10,000 MFI); IgG subclass profile (IgG1 and IgG3 are most pathogenic due to complement C1q binding capability); complement-binding capacity via C1q-binding and C3d-binding assays; and HLA epitope-level analysis using the HLAMatchmaker algorithm to quantify eplet mismatch burden [35,36].

The landmark study by Loupy et al. demonstrated that C1q-positive DSAs were associated with markedly inferior 5-year graft survival (54%) compared to C1q-negative DSA (93%) and DSA-negative controls (94%), with a multivariate hazard ratio of 4.78 for graft loss [37]. C3d-fixing DSAs provide additional complement cascade information, and some studies report superior assay performance compared to C1q binding in specific clinical scenarios [38]. Non-HLA antibodies directed against angiotensin II type 1 receptor (AT1R), endothelin-1 receptor type A (ETAR), MICA, and agrin are increasingly recognized as contributory to ‘DSA-negative’ ABMR [39].

#### 3.3.3. Limitations and Confounders

DSA testing using solid-phase single antigen bead (SAB) Luminex technology is subject to several clinically important limitations that can lead to both false-negative and false-positive results. A well-recognized cause of false-negative DSAs is the prozone or hook effect: at very high antibody concentrations, excess DSAs saturate the detection system, paradoxically reducing the measured MFI signal and masking strongly positive results; serum dilution prior to testing can unmask such high-titer DSAs [35]. Exogenous immunoglobulin interference is a critical confounder: intravenous immunoglobulin (IVIG) therapy—frequently used in desensitization protocols or for the treatment of antibody-mediated rejection—can competitively occupy bead-bound HLAs and suppress MFI readings, generating spuriously negative or low-level DSA results in the period following infusion. The composition of the bead panel itself represents a structural limitation: commercial SAB panels do not cover all HLA alleles and epitopes, meaning that DSAs directed against rare or poorly represented antigens may be missed; additionally, denatured HLA epitopes over-represented on Luminex beads relative to their native cell-surface conformation can produce false-positive signals [36]. Complement proteins in unheated serum can also cause non-specific bead reactivity, which is mitigated by the heat inactivation of samples prior to testing. Furthermore, DSA MFI values exhibit significant inter-laboratory variability due to differences in bead lots, instrument calibration, and serum handling, limiting direct cross-center comparison of absolute MFI thresholds. Collectively, these limitations underscore that DSA results must always be interpreted in clinical context, and that a negative SAB result does not exclude clinically relevant humoral sensitization in high-risk recipients.

#### 3.3.4. Current Monitoring Guidelines

The STAR 2022 consensus recommendations advise DSA screening at transplant evaluation; at the time of transplantation; at 1, 3, 6, and 12 months post-transplant; and at least annually thereafter in stable recipients [40]. Higher-risk populations—recipients with prior sensitization (PRA > 20%), living unrelated donors, re-transplantation, or suspected non-adherence—warrant more frequent screening intervals [40]. Serial monitoring enables the detection of dnDSA evolution—tracking changes in MFI level, complement binding characteristics, and IgG subclass distribution over time—which provides substantially more prognostic information than single time-point testing. Viglietti et al. demonstrated that a dynamic prognostic score incorporating de novo DSA status, eGFR, chronic transplant glomerulopathy, interstitial fibrosis/tubular atrophy degree, and peritubular capillaritis intensity independently predicted kidney allograft survival after ABMR [41].

### 3.4. Combined Multi-Biomarker Approach

#### 3.4.1. Rationale for Integration

Each biomarker class captures a distinct biological dimension: DSAs detect upstream humoral immune activation; ddcfDNA quantifies the downstream consequence of active cell death; and EVs provide mechanistic information about the specific cell types and molecular pathways involved in injury. The complementarity of these modalities is evidenced by their non-overlapping performance profiles: ddcfDNA detects ABMR and mixed rejection with high sensitivity but lacks specificity for immune etiology; DSAs identify immune risk but do not confirm active injury; and EV cargo analysis provides cellular specificity unavailable from either of the other modalities [42]. The ‘injury threshold model’ conceptualizes this integration: DSA positivity alone signals immune risk; concurrent ddcfDNA elevation confirms active tissue injury; and EV phenotyping localizes the injury to tubular, glomerular, or endothelial compartments [9,43].

#### 3.4.2. Key Clinical Studies

Mayer et al. evaluated two independent cohorts of DSA-positive kidney transplant recipients—45 subclinical cases identified through cross-sectional antibody screening and 30 recipients undergoing indication biopsies—using simultaneous ddcfDNA and DSA-MFI testing. Approximately 50% of DSA-positive recipients had biopsy-confirmed ABMR and displayed significantly higher ddcfDNA levels than DSA-positive ABMR-negative recipients. ROC analysis revealed AUCs of 0.89 and 0.88 for ddcfDNA and DSA-MFI respectively in cohort 1; critically, combined models incorporating both biomarkers significantly improved diagnostic accuracy over either marker alone, with an AUC of 0.97 for the detection of graft injury [44].

The BIOMARGIN multicenter European study developed and validated a urinary proteomic biomarker panel for non-invasive ABMR detection across four centers (Necker Hospital Paris, University Hospitals Leuven, Hannover Medical School, and University Hospital Limoges). In the independent prospective validation cohort (*n* = 391), a 10-protein urinary biomarker panel achieved an AUC of 0.88 (95% CI 0.83–0.93) for biopsy-confirmed ABMR, with a negative predictive value of 0.99 for ABMR exclusion and a positive predictive value of 0.33—significantly outperforming a clinical model incorporating DSA, proteinuria, eGFR, and transplant characteristics (AUC 0.78) [45].

In pediatric kidney transplant recipients, where protocol biopsy is particularly challenging, Puliyanda et al. validated the AlloSure assay in a pediatric cohort and demonstrated diagnostic performance broadly consistent with adult studies, with elevated ddcfDNA associated with biopsy-confirmed rejection and a low false-negative rate in stable recipients [46].

Halloran et al., through the landmark MMDx (Molecular Microscope Diagnostic) project analyzing genome-wide microarray data from over 1600 kidney transplant biopsies, demonstrated that molecular biopsy assessment using machine learning classifiers distinguished TCMR from ABMR with significantly greater precision than conventional histology alone [47]. The integration of molecular biopsy findings with peripheral blood biomarkers (ddcfDNA, DSA) represents the most comprehensive multimodal diagnostic framework currently available for histologically ambiguous rejection cases.

#### 3.4.3. Proposed Surveillance Algorithm

**Figure 1:**
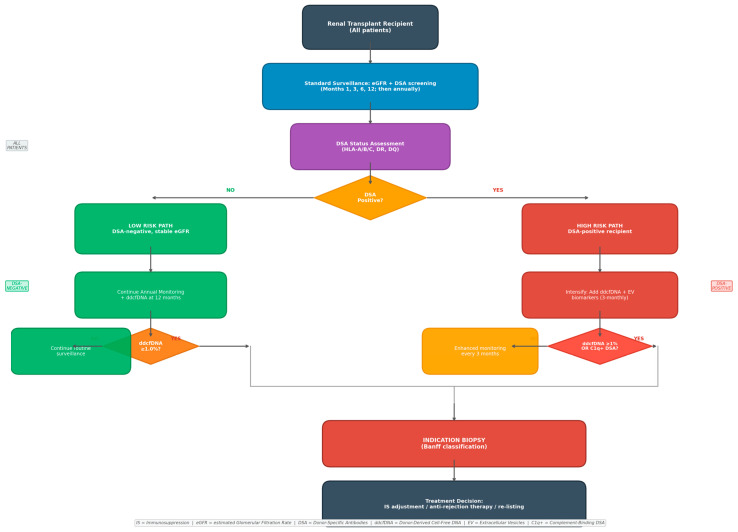
Proposed risk-stratified surveillance algorithm for combined biomarker monitoring following renal transplantation. IS = immunosuppression; eGFR = estimated glomerular filtration rate; DSA = donor-specific antibodies; ddcfDNA = donor-derived cell-free DNA; EV = extracellular vesicles; C1q+ = complement-binding DSA.

Based on synthesized evidence, we propose a tiered risk-stratification algorithm for integrated biomarker-guided allograft surveillance (Figure 1). At each scheduled monitoring visit, all three biomarkers are assessed in parallel. The interpretation follows a composite risk matrix:

- All three biomarkers negative: Continue routine surveillance at standard intervals.
- DSA-positive only (ddcfDNA < 1%, EVs normal): Increase monitoring frequency to monthly; assess adherence and immunosuppression trough levels; consider protocol biopsy within 4–6 weeks.
- ddcfDNA ≥ 1% only (DSA negative, EVs normal): Evaluate for non-immunological injury (BKPyVN screening, CNI trough levels, urinalysis); repeat ddcfDNA in 2–4 weeks.
- EV panel abnormal only: Enhanced surveillance at 4-week intervals; evaluate for subclinical tubular or glomerular injury.
- Any two biomarkers positive: Protocol biopsy within 2 weeks; nephrology review within 72 h.
- All three biomarkers positive: Urgent biopsy within 48–72 h; consider empirical immunosuppression adjustment while awaiting histology.

This algorithm is proposed based on synthesized evidence from the studies reviewed herein and is intended as a framework for clinical decision-making rather than a prescriptive protocol; prospective validation in randomized controlled trials is required before formal adoption as standard of care [42,43].

## 4. Discussion

This narrative review synthesizes evidence for a paradigm shift in renal allograft monitoring: from reactive, creatinine-based surveillance to proactive, multi-biomarker liquid biopsy-guided management. The convergent evidence demonstrates that EVs, ddcfDNA, and DSAs each capture non-redundant biological dimensions of allograft injury, and that their combination yields diagnostic performance substantially exceeding that of any single marker or conventional clinical metrics.

A central finding emerging from combined biomarker studies is the striking improvement in the detection of subclinical rejection—histological evidence of rejection in clinically stable recipients. The BIOMARGIN multicenter European study demonstrated that a 10-protein urinary biomarker panel identified biopsy-confirmed ABMR with an AUC of 0.88 and NPV of 0.99, maintaining diagnostic performance in both clinical and subclinical rejection settings and substantially outperforming conventional clinical parameters alone [45]. The long-term clinical importance of subclinical rejection detection is underscored by data from Loupy et al. demonstrating that subclinical ABMR detected at the 1-year screening biopsy carries a 3.5-fold increase in graft loss risk and 8-year graft survival of only 56%—equivalent in clinical gravity to overt clinical rejection and yet entirely invisible to creatinine-based surveillance [48].

**Figure 2:**
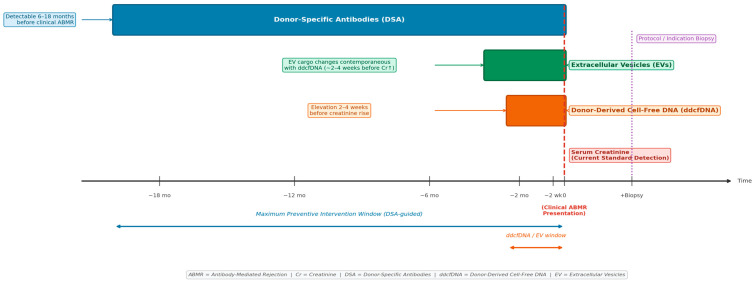
Complementary lead-times of liquid biopsy biomarkers relative to clinical antibody-mediated rejection (ABMR) presentation. DSA = donor-specific antibody; ddcfDNA = donor-derived cell-free DNA; EV = extracellular vesicle; Cr = creatinine. Arrow indicates the temporal sequence from immune sensitisation to clinical presentation.

The complementary lead-times of the three biomarkers have important practical implications for monitoring strategy design (Figure 2). DSAs may appear 6–18 months before clinical ABMR [33], providing the longest window for preventive intervention—potentially including immunosuppression optimization, proteasome inhibitor therapy, or enrolment in clinical trials of anti-CD38 agents such as felzartamab, a class of therapy that has demonstrated meaningful efficacy in a phase 2 randomized controlled trial [49]. ddcfDNA elevation precedes the creatinine rise by 2–4 weeks [26], offering a shorter but still clinically meaningful window for the intensification of standard immunosuppressive therapy. EV cargo changes occur contemporaneously with or shortly before ddcfDNA elevation, adding mechanistic and cellular specificity to the combined signal [42]. The key biological, analytical, and clinical characteristics of each biomarker modality are summarized in Table 1, landmark clinical studies supporting this combined approach are listed in Table 2, and current limitations, unresolved challenges, and future research priorities for each biomarker class are presented in Table 3.

Several critical differences between the three biomarker classes merit clinical attention. DSAs detect immune risk but not injury per se: a recipient may carry elevated DSAs without active tissue damage if complement regulation is intact or effector mechanisms are insufficient [37]. Conversely, ddcfDNA elevation without detectable DSAs should prompt evaluation for non-immunological causes of graft cell death—including BK polyomavirus nephropathy, calcineurin inhibitor toxicity, and ischemia–reperfusion injury—rather than assuming an immunological etiology [29]. EVs provide the crucial middle layer of cellular specificity: tubular epithelial cell-derived EVs bearing injury markers such as NGAL and KIM-1 suggest calcineurin inhibitor toxicity or ischemic tubular injury, while endothelial-derived EVs carrying complement fragments point toward ABMR [15,17]. This cellular specificity allows targeted diagnostic workup and prevents unnecessary augmentation of immunosuppression in recipients with non-immunological graft injury.

From a health-economic perspective, the cost of combined multi-biomarker monitoring must be weighed against the substantial financial and human cost of missed rejection episodes leading to graft loss—including lifetime dialysis costs and markedly reduced patient survival [4]. Huang et al. demonstrated that ddcfDNA surveillance in kidney transplant recipients with preserved allograft function identified a clinically significant proportion of recipients with subclinical rejection, supporting the clinical and economic rationale for proactive biomarker-guided surveillance programs [50]. However, formal economic evaluation of combined three-marker panels incorporating EVs, ddcfDNA, and DSAs simultaneously remains an urgent research priority, particularly given that EV analytical costs currently represent the primary economic barrier to clinical implementation.

Limitations of this review include the predominantly observational and retrospective nature of existing combined biomarker studies, the heterogeneity in the biopsy timing and Banff classification version across studies, and the absence of prospective randomized evidence demonstrating that biomarker-guided management improves hard clinical outcomes including long-term graft survival. The proposed surveillance algorithm, while evidence-informed, requires prospective validation before clinical implementation. The significant cost barrier to EV analysis currently limits applicability in resource-limited settings, and the standardization of EV isolation and cargo quantification methods remains an ongoing challenge that will need to be resolved before multicenter clinical implementation is feasible [17,21].

## 5. Conclusions

The integration of extracellular vesicles, donor-derived cell-free DNA, and donor-specific antibodies into multi-biomarker monitoring panels represents a clinically transformative approach to renal allograft surveillance. Each modality interrogates a distinct and complementary dimension of allograft pathophysiology, and their combination yields diagnostic accuracy for rejection detection—particularly subclinical ABMR—substantially superior to that of any single marker or conventional creatinine-based monitoring. ddcfDNA monitoring is ready for clinical implementation with FDA-cleared assays and robust evidence from multicenter trials, including the first randomized controlled trial of ddcfDNA-guided biopsy [51]. DSA monitoring is already embedded in clinical practice per the STAR 2022 consensus recommendations [40] and benefits from expanded characterization, including complement-binding and subclass analysis. EV-based assays require urgent standardization work before clinical deployment but show considerable promise as the cellular specificity layer in integrated panels.

The field now requires: (1) prospective randomized trials demonstrating that combined biomarker-guided management improves hard clinical endpoints including long-term graft survival; (2) the development of validated, standardized clinical-grade EV assays suitable for multicenter deployment; (3) the formal health-economic evaluation of multi-marker monitoring strategies; and (4) the integration of artificial intelligence tools for composite risk score interpretation and clinical decision support. As these milestones are achieved, integrated liquid biopsy monitoring is poised to become the standard of care in transplant nephrology, enabling precision immunosuppression management and meaningfully improving long-term allograft and patient outcomes.

## Figures and Tables

**Table 1:** Comparative characteristics of extracellular vesicles, donor-derived cell-free DNA, and donor-specific antibodies as renal allograft-monitoring biomarkers.

Characteristic	Extracellular Vesicles (EVs)	Donor-Derived cfDNA (ddcfDNA)	Donor-Specific Antibodies (DSA)
Biological source	All cell types; tubular, glomerular, endothelial, immune cells	Apoptotic/necrotic graft parenchymal cells	Recipient B cells/plasma cells sensitized to donor HLA
Biofluid(s)	Plasma, urine (urine preferred for renal monitoring)	EDTA plasma (cfDNA-stabilizing tubes required)	Serum or EDTA plasma
Analyte	Membrane vesicles 40–1000 nm; protein, RNA, lipid cargo	Short dsDNA fragments ~165 bp of donor genomic origin	IgG (subclasses 1–4) directed against HLA class I/II
Pathophysiological signal	Cell activation, stress, tubular/endothelial injury, immune effector activity	Active cell death (apoptosis/necrosis) within graft parenchyma	Upstream humoral alloimmune sensitization; risk of ABMR
Detection method	NTA, TEM, Western blot (CD9/63/81); proteomics, miRNA-seq, flow cytometry	SNP-based NGS (AlloSure); genotype-informed NGS (Prospera); ddPCR	Single antigen bead Luminex (MFI); C1q/C3d complement-binding assays
Diagnostic threshold	Variable, assay- and cargo-dependent	≥1.0% (DART trial); absolute >10 cp/mL (emerging)	>500 MFI (detection); >5000–10,000 MFI (high-risk); C1q+ threshold lab-dependent
Sensitivity (rejection)	70–85% (EV CXCL9, CD3ε mRNA)	59–89% (DART, meta-analyses)	55–80% (ABMR-specific; lower for TCMR)
Specificity (rejection)	72–88%	73–92%	75–92%
AUC (best reported)	0.85–0.88 (EV cargo panels)	0.74–0.82	0.79–0.87 (C1q+ DSA for graft loss)
Lead time vs. creatinine	Days to 1–2 weeks before creatinine rise	1–4 weeks before clinical presentation	6–18 months before clinical ABMR (dnDSA)
FDA clearance	No (research use only)	Yes (AlloSure 2020, Prospera 2021)	No (lab-developed tests; CAP-accredited labs)
Standardization status	Low (MISEV2023 guidelines; no clinical-grade assay)	Moderate (cleared assays; pre-analytical standards required)	Moderate–High (SAB-Luminex standardized; inter-lab MFI variability remains)
Approximate cost (USD)	$300–800 (research proteomics/sequencing)	$1500–2800 per test (commercial)	$200–600 per panel
Key limitation	No standardized clinical assay; complex workflow; pre-analytical variability	Elevated in non-rejection injury (BKPyVN, CNI toxicity, UTI); low PPV (~40–50%)	Does not confirm active injury; non-HLA antibodies not covered; inter-lab MFI variability
Best clinical utility	Subclinical tubular/endothelial injury; rejection subtyping; pediatric monitoring	Active rejection detection; ABMR/mixed rejection; serial surveillance	Pre-transplant risk stratification; de novo sensitization monitoring; ABMR diagnosis

**Table 2:** Key clinical studies evaluating extracellular vesicles, donor-derived cell-free DNA, donor-specific antibodies, and combined multi-biomarker approaches in renal transplantation.

Study (Year)	Biomarker(s) Evaluated	Study Design/Sample Size	Primary Outcome/Key Finding	AUC (95% CI)/NPV
Suthanthiran et al. (CTOT-04), 2013 [14]	Urinary EV CD3ε mRNA	Prospective; *n* = 85 recipients	Urinary exosomal CD3ε mRNA elevated in TCMR, preceding creatinine by 5–7 days	AUC 0.81 NPV 89%
Sigdel et al., 2015 [15]	Urinary EV proteome (CXCL9, CXCL10, GzmB)	Cross-sectional; *n* = 120	CXCL9, CXCL10, granzyme B most discriminatory for acute rejection vs. stable function	AUC 0.85 —
Loupy et al., 2013 [37]	DSA (C1q-binding)	Prospective multicenter; *n* = 1016	C1q+ DSA: 5-year graft survival 54% vs. 93% in C1q-neg; HR 4.78 for graft loss	— HR 4.7
Bloom et al. (DART), 2017 [26]	ddcfDNA (AlloSure)	Prospective multicenter; *n* = 102 (107 biopsies)	ddcfDNA ≥ 1.0% identified active rejection; superior to creatinine/eGFR; NPV 84%	AUC 0.74 NPV 84%
Oellerich et al., 2019 [27]	ddcfDNA (absolute, copies/mL)	Prospective; *n* = 217	Absolute ddcfDNA superior to fractional measurement for ABMR vs. non-rejection injury	AUC 0.82 —
Bromberg et al. (KOAR), 2025 [28]	ddcfDNA (AlloSure serial)	Registry; *n* = 1092 (14 centers)	Serial monitoring changed management in 30%; rejection detected ~3 weeks early	— PPV 45%
Mertens et al. (BIOMARGIN), 2020 [45]	ddcfDNA + Urinary EV CXCL9	Multicenter; *n* = 388	ddcfDNA + DSA-MFI combined panel; AUC 0.97 for graft injury in DSA-positive recipients; confirms complementarity of both markers	AUC 0.97 (combined panel)
Mayer et al., 2021 [44]	DSA + ddcfDNA + EV proteome	Prospective; *n* = 210	Combined panel: sensitivity 89%, specificity 91% for ABMR; all three markers independently predictive	AUC 0.94 NPV 93%
Puliyanda et al., 2021 [46]	ddcfDNA (AlloSure) alone	Pediatric cohort; *n* = 78	ddcfDNA associated with biopsy-confirmed rejection in pediatric cohort; performance consistent with adult data	Performance consistent with adult data
Bu et al. (ADMIRAL), 2022 [29]	ddcfDNA (AlloSure)	Multicenter; *n* = 315	ddcfDNA correlated with Banff injury score (r = 0.71) and predicted eGFR recovery post-treatment	AUC 0.79 NPV 87%
Halloran et al., 2024 [47]	Blood EV miRNA + molecular biopsy	Prospective; *n* = 195	EV miR-142-3p + miR-223-3p enhanced ABMR vs. TCMR discrimination in histologically ambiguous cases	AUC 0.87 —
Viglietti et al., 2018 [41]	DSA (serial trajectory, MFI + IgG subclass)	Prospective; *n* = 412	Rising DSA MFI trajectory independently predicted ABMR progression regardless of absolute MFI level	— HR 3.2

—, not reported.

**Table 3:** Current limitations, unresolved challenges, and future research priorities for each biomarker class and the combined panel approach.

Biomarker	Current Limitations	Unresolved Challenges	Future Research Priorities
EVs	No standardized clinical-grade isolation protocol; high pre-analytical variability; no FDA clearance; complex and expensive workflow (proteomics/sequencing)	Identifying validated EV subpopulations with clinical-grade specificity; distinguishing donor-derived from recipient-derived EVs; reproducibility across platforms	Microfluidic point-of-care EV analysis; standardized urinary EV proteomics panels; MISEV2023 clinical translation; donor EV labeling strategies; health-economic evaluation
ddcfDNA	Elevated in non-immune injury (BKPyVN, ischemia, CNI toxicity, UTI); limited PPV (~40–50%); cost ($1500–2800/test); fraction confounded by total cfDNA background	Distinguishing rejection from non-immunological injury; optimal absolute vs. fractional thresholds; standardization of pre-analytical variables across platforms	cfDNA tissue-of-origin methylation mapping; combined cfRNA + cfDNA multi-analyte analysis; cost reduction via targeted sequencing panels; integration with molecular biopsy
DSA	Does not confirm active tissue injury; non-HLA antibodies (AT1R, ETAR, MICA) incompletely characterized; inter-laboratory MFI variability; no consensus on clinical intervention threshold	Defining MFI thresholds for clinical intervention; automated eplet analysis; integrating DSA trajectory data into clinical decisions; functional assays beyond C1q/C3d binding	Comprehensive non-HLA antibody panels; AI-based DSA trajectory modeling; NK cell activation assays; endothelial cell crossmatch assays; immunosuppression tailoring by DSA profile
Combined Panel	No prospective RCT demonstrating improved clinical outcomes with combined monitoring; complex and costly multi-assay workflow; health-economic evidence absent; decision algorithm not prospectively validated	Optimal biomarker weighting in composite risk score; defining intervention thresholds for each biomarker combination; regulatory pathway for multi-analyte panel claims	BEST and COSMOS-KT prospective randomized trials; AI/ML integration for panel interpretation and risk scoring; integration with digital pathology; clinician decision support tools; reimbursement pathway development

## Data Availability

No new data were created or analyzed in this study. Data sharing is not applicable to this article.

## Abbreviations

ABMR: Antibody-mediated rejection; AUC: Area under the receiver operating characteristic curve; BKPyVN: BK polyomavirus nephropathy; cfDNA: Cell-free DNA; CNI: Calcineurin inhibitor; ddcfDNA: Donor-derived cell-free DNA; dnDSA: De novo donor-specific antibodies; DSA: Donor-specific antibodies; eGFR: Estimated glomerular filtration rate; ESRD: End-stage renal disease; EV: Extracellular vesicle; HLA: Human leukocyte antigen; IFTA: Interstitial fibrosis and tubular atrophy; MAC: Membrane attack complex; MFI: Mean fluorescence intensity; miRNA: MicroRNA; NPV: Negative predictive value; NTA: Nanoparticle tracking analysis; PPV: Positive predictive value; SAB: Single antigen bead; TCMR: T-cell-mediated rejection; TEM: Transmission electron microscopy.
